# ERK1/2 Signaling Pathway Activated by EGF Promotes Proliferation, Transdifferentiation, and Migration of Cultured Primary Newborn Rat Lung Fibroblasts

**DOI:** 10.1155/2020/7176169

**Published:** 2020-10-05

**Authors:** Yu Hu, JianHua Fu, XueYan Liu, XinDong Xue

**Affiliations:** Department of Pediatrics, Shengjing Hospital of China Medical University, 36 Sanhao Street, Shenyang, Liaoning 110004, China

## Abstract

**Background:**

Bronchopulmonary dysplasia (BPD) is a common and serious complication in premature infants. Lung fibroblasts (LFs) are present in the extracellular matrix and participate in pulmonary development in response to BPD. The aim of this study was to investigate the effect of extracellular signal-regulated kinase (ERK) on LFs cultured from newborn rats. *Material and Methods*. Primary LFs were isolated and treated with epidermal growth factor (EGF, 20 ng/mL) in the presence or absence of an ERK inhibitor, PD98059 (10 *μ*mol/L). Phosphorylated ERK1/2 (p-ERK1/2) protein levels were determined using immunocytochemistry, western blotting, and real-time reverse transcription quantitative (RT–q)PCR. LF proliferation was examined by flow cytometry and a cell counting kit-8 assay. LF transdifferentiation was examined by protein and mRNA expression of *α*-smooth muscle actin (*α*-SMA) by immunocytochemistry, western blotting, and RT–qPCR. LF migration was examined by the transwell method.

**Results:**

Phosphorylated ERK1/2, which was activated by EGF, promoted LF proliferation by accelerating cell-cycle progression from the G1 to S phase. After treatment with PD98059, the expression of p-ERK1/2 in LFs, cellular proliferation, and the percentage of cells in S phase were significantly decreased. Phosphorylated ERK1/2 also promoted the differentiation of LFs into myofibroblasts through increased *α*-SMA synthesis and migration.

**Conclusion:**

The activation of ERK promotes proliferation, transdifferentiation, and migration of lung fibroblasts from newborn rats.

## 1. Introduction

Bronchopulmonary dysplasia (BPD) is a serious chronic pulmonary complication often seen in preterm neonates experiencing long-term ventilation support or oxygen exposure [1]. The morbidity costs of this disease include a more than 30% increase in the number of extra low birth-weight infants born each year in the USA, with mortality as high as 10~25% [2, 3]. Despite advances in the prevention and therapy of respiratory distress syndrome in premature infants, BPD remains a major disease, with many infants experiencing significant recurrent respiratory tract infections and airway hyperresponsiveness throughout childhood [4, 5]. During normal lung development and differentiation, epithelial and mesenchymal cells ordinarily proliferate and interact. The interruption of such processes may impact lung structure and function and cause disastrous consequences [6].

The main pathological features of BPD are simplified enlarged alveoli, capillary dysplasia, and pulmonary interstitial fibrosis [7]. Although most studies have focused on alveolar epithelial cells in BPD, lung fibroblasts (LFs) also participate in normal lung development and respond to lung injury. Alveolar development and repair are affected by the neighboring pulmonary interstitial fibroblasts, which, in turn, affect extracellular matrix (ECM) remodeling and can cause fibrosis [8]. The main manifestation of pulmonary interstitial fibrosis is the uncontrolled growth of lung fibroblasts that are deposited in the pulmonary interstitium or displaced normal lung epithelium [9].

Pulmonary mesenchymal and epithelial cells interact during localized branching, growth, and extension of primordial tubules. Disruption of this process in premature infants exposed to hyperoxia contributes to BPD [10]. The pathological characteristics of BPD include an uneven proliferation of mesenchymal LFs [8]. The regulation of lung fibroblast proliferation is, thus, of clinical and developmental interest.

The extracellular signal-regulated kinase (ERK) cascade plays a vital role in cellular proliferation, especially in tumorigenesis [11]. We have previously found that hyperoxia promoted ERK1/2 phosphorylation and the proliferation of lung fibroblasts rats *in vitro* [12]. However, the relationship between ERK1/2 and lung fibroblasts is not clear. Fibroblasts act as secretory cells in the lung tissue and mediate normal and pathological remodeling during pulmonary development. However, to date, the role of ERK signaling in cultured primary LFs has been poorly studied. Consequently, in this study, we evaluated mechanisms of cell proliferation, transdifferentiation, and migration of cultured primary lung fibroblasts treated with the ERK activator, epidermal growth factor (EGF), in the presence or absence of the inhibitor, PD98059. Our particular focus was on the impact of activating p-ERK1/2 on proliferation, transdifferentiation, and migration of lung fibroblasts, and the molecular mechanism(s) by which activation of ERK1/2 modulates the progression of BPD.

## 2. Materials and Methods

### 2.1. Culturing and Treatment of Primary Lung Fibroblasts

The study was approved by the institutional research ethics committee of China Medical University. Newborn rat pups on day 3 of the saccular stage (*n* = 30) were euthanized with an intraperitoneal injection of sodium pentobarbital (l00 mg/kg) prior to removing the lung tissue. When the heart stopped beating and the pupils became dilated, the lung tissue was removed under sterile conditions, finely minced, and digested with 0.25% trypsin (Santa Cruz Biotechnology, Santa Cruz, CA, USA) in a shaking water bath for 30 min at 37°C. Cells were collected and cultured in Dulbecco's modified Eagle's medium (DMEM; Thermo Scientific HyClone, Beijing, China) with 10% fetal bovine serum (FBS; Thermo Scientific HyClone, Logan, UT, USA). LFs were used in experimental procedures when they became confluent, and showed a typical elongated and spindle-shaped appearance. LFs were identified by vimentin (sc-5565, 1 : 100; Santa Cruz Biotechnology, Santa Cruz, CA, USA) staining as we have published previously [13].

LFs were randomly divided into four groups: (1) untreated control; (2) EGF group, which was treated with 20 ng/mL EGF (Peprotech, Rocky Hill, NJ, USA); (3) PD98059 group, which was treated with 10 *μ*mol/L PD98059 (Cell Signaling Technology, Boston, MA, USA); and (4) EGF+PD98059 group, which was treated with 20 ng/mL EGF and 10 *μ*mol/L of the ERK inhibitor, PD98059. LFs were collected following a 24-h treatment.

### 2.2. Immunocytochemistry

LFs were fixed in paraformaldehyde. The proteins, p-ERK1/2 and *α*-smooth muscle actin (*α*-SMA), were detected using standard streptavidin–peroxidase immunocytochemical staining procedures. Cells were incubated with primary antibody (#4370 for p-ERK1/2 and #56856 for *α*-SMA, both 1 : 100; Cell Signaling Technology, Boston, MA, USA) at 4°C overnight. The same procedure without the primary antibody was used as an isotype control. Cells were incubated with secondary antibody (SP-9000; Beijing Zhongshan Goldenbridge Biotechnology, Beijing, China) for 20 min at 37°C, followed by horseradish peroxidase (HRP-) labeled streptavidin at 37°C for 20 min. Cells were washed with PBS three times between steps. After diaminobenzidine (DAB) staining, LFs were observed using a MetaMorph Imaging System (Universal Imaging Corporation, West Chester, PA, USA). The optical density value was semiquantitatively measured using the MetaMorph software. Five randomly selected high-power fields (400x magnification) were photographed for each slide.

### 2.3. Western blotting of ERK, p-ERK, Type *Ι* collagen, and *α*-SMA in LFs

Total protein was extracted from LFs, quantified, separated by SDS-polyacrylamide gel electrophoresis, and then transferred to nitrocellulose membranes. Membranes were blocked for 1 h with 5% nonfat milk followed by incubation with anti-p-ERK1/2, anti-ERK, anti-type *Ι* collagen (Col-*Ι*), or anti-*α*-SMA primary antibody (#4370 for p-ERK1/2, #4696 for ERK1/2, #91144S for Col-*Ι*, #56856 for *α*-SMA; all 1 : 1000; Cell Signaling Technology, Boston, MA, USA) overnight at 4°C, and then with HRP-labeled secondary antibody (ZB2305, 1 : 2000; Beijing Zhongshan Goldenbridge Biotechnology, Beijing, China) at 37°C for 2 h. The blots were developed using electrochemiluminescence after washing three times with Tris-buffered saline containing Tween 20. Films were digitized, and relative grayscale values were calculated. The higher the relative grayscale value, the higher the protein level.

### 2.4. Cell-Cycle Analysis by Flow Cytometry

To arrest cells in the G0 phase in the cell cycle, LFs were cultured for 24 h in serum-free DMEM, divided into four treatment groups, and then cultured in DMEM with 10% FBS for 24 h. LFs were harvested, suspended (1 × 10^6^ cells/mL), then fixed in 75% cold ethanol at 4°C. The LFs were treated with 50 *μ*g/mL RNase (Sigma, St. Louis, MO, USA) at 37°C for 30 min then stained with 100 *μ*g/mL propidium iodide (Sigma) at 4°C for 30 min in the dark. The distribution of the cell cycle was evaluated by flow cytometry (Becton, Dickinson and Company, Franklin Lakes, NJ, USA).

### 2.5. CCK-8 Colorimetric Assay

LFs were cultured for 24 h in serum-free DMEM and then divided into the four treatment groups in 24-well plates (5 × 10^5^ cells/100 *μ*L/well). LFs were incubated for 6, 12, or 24 h with EGF, PD98059, or EGF+PD98059 at 37°C. Cell counting kit-8 (CCK-8; #C0038; Beyotime, Shanghai, China) was added to each well (10 *μ*L/well) for the final 1 h. The absorbance was calculated at 450 nm by enzyme-linked immunosorbent assay reader (Shimadzu UV-260, Kyoto, Japan).

### 2.6. Real-Time Reverse Transcription Quantitative PCR

Quantitative changes in ERK1, ERK2, and *α*-SMA expression were determined by real-time reverse transcription-quantitative (RT–q)PCR. TRizol reagent (Takara, Shiga, Japan) was used to extract total RNA. Aliquots (500 ng/*μ*L) were reverse transcribed to cDNA for 15 min at 37°C and then 5 sec at 85°C with a PrimeScript RT Reagent Kit (DRR037S; Takara Biotechnology, Dalian, China). Real-time reverse transcription-quantitative PCR assay conditions were 40 cycles for 10 sec at 95°C, 40 cycles for 5 sec at 95°C, followed by 20 sec at 60°C using SYBR^R^*Premix Ex Taq*™ (DRR041S; Takara Biotechnology, Dalian, China) and a LightCycler 2.0 RT–qPCR System (Roche Diagnostics, Indianapolis, IN, USA). Gene-specific primers were designed and synthesized by Takara. Specific primers were used to detect ERK1, ERK2, *α*-SMA, and GAPDH (Table 1). For each sample, a *Δ*Ct value was obtained.

### 2.7. Cell Migration Assay

Cellular migration is necessary for the extracellular matrix process. To quantify cell movement, a simple chamber setup was utilized to assess migration in response to different treatment stimuli. A simple Boyden chamber is made up of an outer wall consisting of plate wells and a transwell insert (8 *μ*m pore diameter) placed within each well. LFs (1 × 10^5^/100 *μ*L/well) were cultured in the top chamber (Transwell) with 600 *μ*L culture solution in the bottom reservoir. LFs were then incubated with EGF, PD98059, or EGF+PD98059 at 37°C for 24 h. The control group was untreated. After the treatment, each membrane was washed with PBS three times and fixed with 4% paraformaldehyde for 30 min. Cells were stained for 15 min using trypan blue and counted.

### 2.8. Statistical Analysis

All experiments were repeated five times. The results are expressed as mean ± standard error (SE). Data were analyzed with ANOVA followed by Student–Newman–Keuls test for multiple comparisons among groups. *P* < 0.05 was considered statistically significant.

## 3. Results

### 3.1. Exposure to EGF Leads to ERK Activation

**Figure 1:**
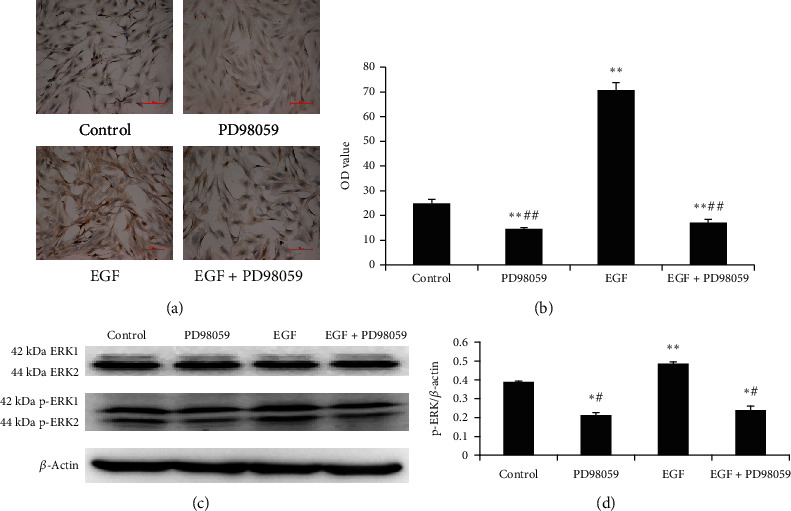
(a, b) Protein expression level of p-ERK was detected by immunocytochemistry in the lung fibroblasts (LFs) following treatment (scale bar = 100 *μ*m). Positive phosphorylated extracellular signal-regulated kinase (p-ERK)1/2 antigen expression was found distributed in the cell cytoplasm and nucleus. (c, d). ERK and p-ERK protein levels in LFs following treatment were detected in the western blots of protein samples from the four groups. Control group: LFs without any treatment; PD98059 group: LFs treated with 10 *μ*mol/L PD98059; epidermal growth factor (EGF) group: LFs treated with 20 ng/mL EGF; EGF+PD98059 group: LFs treated with both 20 ng/mL EGF and 10 *μ*mol/L PD98059. All treatments were administered for 24 h. Data are presented as the mean ± standard deviation (SD; *n* = 5). ^∗^*P* < 0.05 vs. control cells. ^∗∗^*P* < 0.01 vs. control cells. ^#^*P* < 0.05 vs. EGF group. ^##^*P* < 0.01 vs. EGF group.

To evaluate pathologic changes in response to EGF, the expression of p-ERK1/2 was examined by immunocytochemistry and western blotting. The immunocytochemical staining of primary LFs demonstrated p-ERK1/2 in the nucleus and cytoplasm (Figure 1(a)). The levels of p-ERK1/2 were significantly higher in the EGF group than those in the control group (*P* < 0.01). PD98059 decreased ERK phosphorylation in LFs (*P* < 0.01). The expression of p-ERK1/2 was decreased in the EGF+PD98059 group compared with that in the EGF group (*P* < 0.01; Figure 1(b)).

Equivalent amounts of cellular proteins in LFs were probed by western blot analysis using a specific p-ERK1/2 antibody. As shown in Figures 1(c) and 1(d), a significant increase in p-ERK1/2 levels in LFs exposed to EGF compared to the control group was observed (*P* < 0.01), which decreased when LFs were cotreated with EGF and PD98059 (*P* < 0.05). PD98059 alone also affected the phosphorylation of ERK1/2 (*P* < 0.05). None of the treatments changed the level of nonphosphorylated ERK1/2 (*P* > 0.05).

Real-time reverse transcription-quantitative PCR also revealed no difference in mRNA levels of ERK1 and ERK2 in each group as listed in Table 2 (all *P* > 0.05).

### 3.2. Activation of ERK Induces Proliferation of Lung Fibroblasts

**Figure 2:**
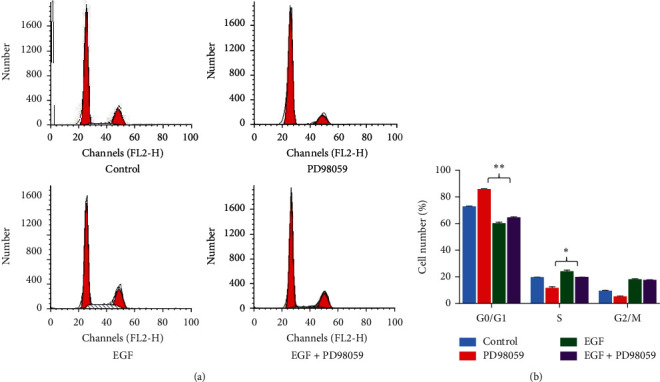
Cell cycle distribution. Cell populations at G0/G1, S, and G2/M phases were determined by averaging the results from five independent experiments. Control group: lung fibroblasts (LFs) without any treatment; PD98059 group: LFs treated with 10 *μ*mol/L PD98059; epidermal growth factor (EGF) group: LFs treated with 20 ng/mL EGF; EGF+PD98059 group: LFs treated with both 20 ng/mL EGF and 10 *μ*mol/L PD98059. All treatments were administered for 24 h. ^∗^*P* < 0.05, ^∗∗^*P* < 0.01 vs. control cells.

The effect of p-ERK1/2 on the proliferation of LFs was investigated by cell-cycle distribution. For the control group, the greatest proportion of cells existed in the G1 phase (Figure 2). Markedly decreased G1-phase and increased S-phase cell populations appeared after EGF treatment for 24 h (*P* < 0.01), indicating that LFs were not arrested at the G1 checkpoint. In contrast, PD98059 significantly increased the G1 phase (*P* < 0.01) and decreased the S-phase cell population (*P* < 0.05). The progression of S-phase cells cotreated with PD98059 and EGF was also significantly slower than that of cells treated only with EGF (*P* < 0.05). These results were in accordance with the p-ERK level, which indicated that activated ERK is essential for cells to enter the S phase.

**Figure 3:**
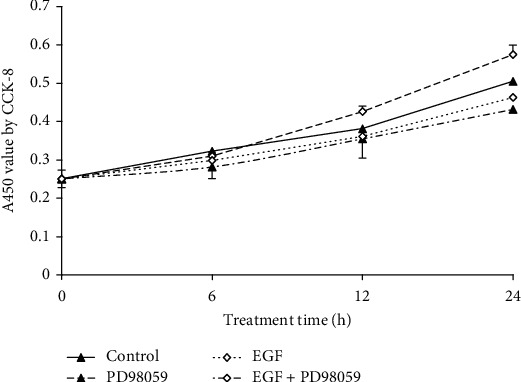
Effect of treatments on LF cell proliferation. The CCK-8 assay was used to measure lung fibroblast (LF) proliferation. Epidermal growth factor (EGF) alone (20 ng/mL) promoted cell proliferation after 24 h under basal conditions (*P* < 0.05). PD98059 treatment significantly inhibited LF cell proliferation from 12 to 24 h (both *P* < 0.05). The coincubation of PD98059 and EGF inhibited the activity of EGF alone in cell proliferation after 12 h (*P* < 0.05) and 24 h (*P* < 0.01).

A CCK-8 assay revealed that the PD98059 treatment significantly inhibited cell proliferation starting at 12 h, with a peak after 24 h (*P* < 0.05). A significant difference between the untreated control and the PD98059 groups for cell proliferation after 12 and 24 h under basal conditions was not noted. The number of LF cells was 1.14-fold higher following the EGF stimulation for 24 h compared to the untreated control group (*P* < 0.05). Cotreatment with PD98059 and EGF resulted in a decrease after 6, 12, and 24 h in the number of LFs by 3.6% (*P* > 0.05), 15.2% (*P* < 0.05), and 19.5% (*P* < 0.01), respectively, compared to the control group (Figure 3).

**Figure 4:**
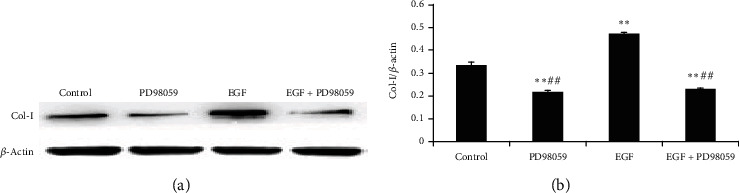
Col-*Ι* protein levels in lung fibroblasts following treatment were detected by western blotting. Control group: lung fibroblasts (LFs) without any treatment; PD98059 group: lung fibroblasts (LFs) treated with 10 *μ*mol/L PD98059; epidermal growth factor (EGF) group: LFs treated with 20 ng/mL EGF; EGF+PD98059 group: LFs treated with both 20 ng/mL EGF and 10 *μ*mol/L PD98059. All treatments were administered for 24 h. Data are presented as the mean ± standard deviation (SD; *n* = 5). ^∗∗^*P* < 0.01 vs. control cells. ^##^*P* < 0.01 vs. EGF group.

Col-I protein was assayed by western blotting. EGF promoted the production of Col-I protein (*P* < 0.01); PD98059 significantly inhibited the effect of EGF (*P* < 0.01) compared with the controls (Figures 4(a) and 4(b)).

### 3.3. Activation of ERK Promotes Transdifferentiation of Lung Fibroblasts

**Figure 5:**
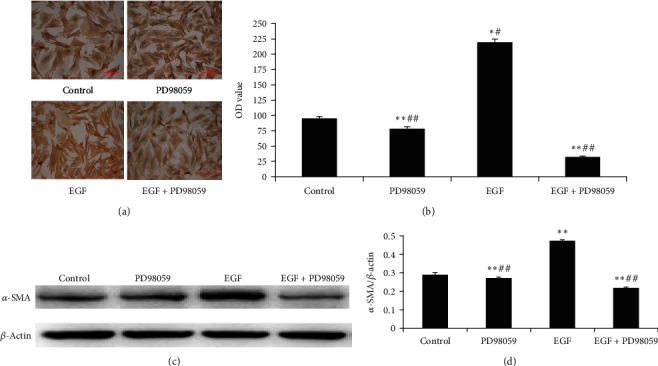
Effect of treatments on alpha smooth muscle actin (*α*-SMA) protein expression. (a, b) *α*-SMA protein expression by immunocytochemical staining and analysis of protein level. (c, d) *α*-SMA protein levels in lung fibroblasts (LFs) following the treatment were detected by western blotting. Control group: LFs without any treatment; PD98059 group: LFs treated with 10 *μ*mol/L PD98059; epidermal growth factor (EGF) group: LFs treated with 20 ng/mL EGF; EGF+PD98059 group: LFs treated with both 20 ng/mL EGF and 10 *μ*mol/L PD98059. All treatments were administered for 24 h. Data are presented as the mean ± standard deviation (SD; *n* = 5). ^∗^*P* < 0.05 vs. control cells. ^∗∗^*P* < 0.01 vs. control cells. ^##^*P* < 0.01 vs. EGF group. Scale bar = 100 *μ*m.

As shown in Figures 5(a) and 5(b), immunocytochemical staining and western blotting of primary LF cultures demonstrated that the *α*-SMA protein expression was significantly increased in the EGF group compared with the control group (*P* < 0.01). This effect was inhibited by PD98059 (*P* < 0.01).

As shown in Table 3, RT–qPCR revealed that the *α*-SMA expression in the EGF group was 2.85-fold higher compared to that in the untreated control group (*P* < 0.01). The expression of *α*-SMA mRNA in the EGF group was significantly inhibited by PD98059 (*P* < 0.01). PD98059 alone also decreased the expression of the *α*-SMA mRNA level compared to the control group (*P* < 0.01).

### 3.4. Activation of ERK Promotes Migration of Lung Fibroblasts

**Figure 6:**
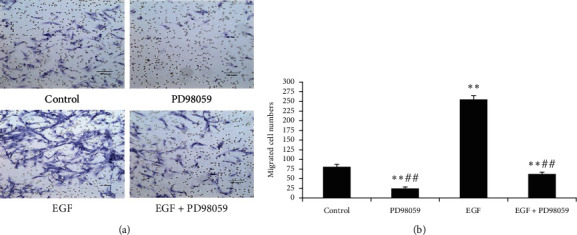
Effects of treatment on the migration of lung fibroblasts using the transwell method. Control group: lung fibroblasts (LFs) without any treatment; PD98059 group: LFs treated with 10 *μ*mol/L PD98059; epidermal growth factor (EGF) group: LFs treated with 20 ng/mL EGF; EGF+PD98059 group: LFs treated with both 20 ng/mL EGF and 10 *μ*mol/L PD98059. All treatments were administered for 24 h. ^∗∗^*P* < 0.01 vs. control cells. ^##^*P* < 0.01 vs. EGF group. Scale bar = 100 *μ*m.

As shown in Figure 6, transwell migration assays revealed that EGF significantly promoted the migration of LFs (*P* < 0.01). Moreover, the expression of p-ERK1/2 was promoted by EGF in LFs. Primary cultured LFs were treated with both EGF and PD98059 for 24 h. The effect of EGF on the LF migration was inhibited by PD98059 (*P* < 0.01).

## 4. Discussion

A newborn's immature pulmonary response to pulmonary surfactants, assisted ventilation, and oxygen supplementation is characterized by a serious injury to alveoli, interstitial tissues, and pulmonary vasculature. This results in tissue remodeling, developmental disorders, and interstitial fibrosis that may ultimately lead to the development of BPD [14, 15]. This pathology is clearly observed in the immature lung cells of newborn rats that develop BPD [16]. Using hematoxylin–eosin staining and immunohistochemistry methods, we have found that the immature lungs exposed to long-term hyperoxia result in fibrogenesis and a significantly higher expression of p-ERK protein in both lung tissue and primary cultured fibroblasts of newborn Wistar rats [12]. In this study, fibroblasts isolated from newborn rat lungs proliferated and migrated following the p-ERK activation after the EGF treatment. This suggests that the activation of ERK1/2 is closely linked to the proliferation, transdifferentiation, and migration of LFs and may play an important role in disturbing the repair or remodeling of pulmonary epithelial cells, giving rise to BPD. Our results were consistent with the pathological changes observed in BPD characterized by interstitial fibrosis.

In the ECM, LFs play essential roles in the response to the pulmonary epithelial injury and interstitial fibrosis in BPD. Several research groups have investigated the effects of the exposure of LFs to hyperoxia, but the results were inconsistent. Lang et al. demonstrated an increased p-ERK1/2, Col-I, and *α*-SMA expression when human fetal LFs were exposed to 95% hyperoxia for 12 h to 48 h [17]. However, two other studies showed conflicting results for primary cultures of fibroblasts obtained from mature adult lungs or in the exposure of cultured fetal rat lung fibroblasts to elevated levels of O_2_, including the inhibition of fibroblast proliferation [18, 19]. Differences in the results obtained may be due to differences in the experimental conditions used and/or observation times. EGF is a soluble factor important for lung development that is active during the epithelial–mesenchymal crosstalk [20]. It acts via the ERK pathway [21–23]. We therefore used EGF to stimulate the activated ERK and found a role for the ERK signaling pathway in regulating LF proliferation.

Hyperoxia activates ERK in neonatal rats on postnatal days 7 and 21 and promotes alveolar interstitial fibroblast transdifferentiation into myofibroblasts [24, 25]. Indeed, the activation of the ERK pathway has been previously demonstrated in lung epithelial cells exposed to hyperoxia, which prolonged cell survival and protected cells from hyperoxia-induced death [26]. Bleomycin and high tidal volume ventilation-induced lung fibrosis depended, in part, on the activation of ERK1/2 pathways. This effect was abrogated by the ERK1/2 inhibitor, PD98059, in mice treated with high tidal volume ventilation [27]. However, the relationship between ERK phosphorylation and BPD development requires further investigation. The present study demonstrated that the stimulation with EGF activated the ERK signaling pathway. This suggests that the underlying molecular mechanisms may involve activated ERK in LFs inducing proliferative and migratory signals.

In addition, the mechanisms by which the ERK pathway acts in BPD may be due to how it participates in normal lung development. The *Mek* gene was recently shown to encode mitogen-activated protein kinase (MAPK), which phosphorylates and activates ERK. A certain amount of *Mek* gene was indispensable to normal placenta development and embryo survival [28]. The *Mek* gene has two homologs: *Mek1* and *Mek2*. Loss of the *Mek1* gene resulted in embryo defects and death while the *Mek2* gene was not necessary because of the compensatory function of the *Mek1* gene [29]. The knockdown of both *Mek* genes in lung epithelial cells resulted in lung hypoplasia and even death. A deficiency of *Mek* genes impaired lung development and caused branching defects in cultured whole-lung embryo explants. The mechanisms involved may be decreased respiratory epithelial cell proliferation coupled with increased cell death [30]. *Mek* gene inactivation in the lung mesenchyme caused lung hypoplasia and tracheal defects because of increased apoptosis and reduced branching. Moreover, it indicated that ERK potentially interacts with the Wnt pathway during lung development [31]. The appearance of pulmonary dysplasia is characterized by reduced branching, increased apoptosis, and decreased mesenchymal cell proliferation.

ERK may also promote fibrosis through transduction signaling by transforming growth factor (TGF)-*β* and matrix metalloproteinases (MMPs). TGF-*β*1 induces collagen production and *α*-SMA expression in human LFs by promoting ERK1/2 activation [32]. The stimulation of TGF-*β* increased MMP and p-ERK1/2 expression in primary cultured human LFs [33]. As a sulfated oligosaccharide that is extracted from seaweed, MS80 arrested the proliferation of human embryonic LFs, MMP activity, and collagen deposition stimulated by TGF-*β*1, and ultimately inhibited bleomycin-induced lung fibrosis in rats [34]. It seems that the formation of fibrosis promoted by MMPs or TGF-*β* is closely related to the activity of ERK1/2. However, direct evidence showing ERK1/2 was upstream or downstream of TGF-*β* or MMP signaling pathways was absent. *In vitro*, it was demonstrated that both rosiglitazone and hydrogen sulfide suppress migration, proliferation, and the phenotypic differentiation of human LFs by inhibiting ERK activation. The two drugs may be effective in patients with pulmonary fibrosis [35, 36]. Above all, ERK1/2 may be a target for the prevention and therapy of pulmonary hypoplasia and fibrosis associated with BPD in premature infants.

## 5. Conclusions

The activation of ERK promotes the proliferation, transdifferentiation, and migration of lung fibroblasts in newborn rats. However, further and more detailed studies are required into the exact cellular pathways involved in the ERK activation in BPD.

## Figures and Tables

**Table 1:** Primers employed for RT–qPCR.

Gene	Sequence	Product size
*ERK1*	Sense 5′-GGTAGACGGTTCTGGAATGGAAGG-3′ Antisense 5′-GTCAGGGAAAATGGGGTGGG-3′	128 bp
*ERK2*	Sense 5′-TGTTCCCAAACGCTGACTCCA-3′ Antisense 5′-AGTCGTCCAGCTCCATGTCAAACT-3′	180 bp
*α*-*SMA*	Sense 5′-AGCCAGTCGCCATCAGGAAC-3′ Antisense 5′-CCGGAGCCATTGTCACACAC-3′	90 bp
*GAPDH*	Sense 5′-GCACCGTCAAGGCTGAGAAC-3′ Antisense 5′-ATGGTGGTGAAGACGCCAGT-3′	142 bp

Notes: ERK: extracellular signal-regulated kinase; GAPDH: glyceraldehyde 3-phosphate dehydrogenase; RT–qPCR: real-time reverse transcriptase quantitative PCR.

**Table 2:** The relative mRNA level of ERK1/2 in LFs $\left(\bar{X}\pm s,n=5\right)$.

△Ct∕group	Control	PD98059	EGF	EGF+PD98059
ERK1	5.16 ± 0.41	5.34 ± 0.36	5.49 ± 0.43	5.48 ± 0.35
ERK2	4.44 ± 0.34	4.50 ± 0.38	4.38 ± 0.24	4.36 ± 0.28

Notes: EGF: epidermal growth factor; ERK: extracellular signal-regulated kinase ^∗^*P* < 0.05 vs. control cells, ^∗∗^*P* < 0.01 vs. control cells.

**Table 3:** The relative mRNA level of *α*-SMA in LFs $\left(\bar{X}\pm s,n=5\right)$.

Group	Control	PD98059	EGF	EGF+PD98059
△Ct value	−1.05 ± 0.29	0.72 ± 0.17^∗∗^	−2.56 ± 0.18^∗∗^	−0.08 ± 0.16^∗∗^

Notes: LF: lung fibroblasts; *α*-SMA: *α*-smooth muscle actin. ^∗∗^*P* < 0.01 vs. control cells.

## Data Availability

All relevant data are within this manuscript, and all data are fully available without restriction.
